# Comparison of the effect of naproxen, etodolac and diclofenac on postoperative sequels following third molar surgery: A randomised, double-blind, crossover study

**DOI:** 10.4317/medoral.19518

**Published:** 2013-12-07

**Authors:** Nihat Akbulut, Evren Üstüner, Cemal Atakan, Gülümser Çölok

**Affiliations:** 1 Assistant Professor; PhD, Department of Oral and Maxillofacial Surgery, School of Dentistry, Gaziosmanpaşa University, Tokat, Turkey; 2 Md, Department of Radiology, School of Medicine, Ankara University, Ankara, Turkey; 3 PhD, Associate Professor, Department of Statistic, Faculty of Sciences, Ankara University, Ankara, Turkey; 4 PhD, Professor, Department of Oral and Maxillofacial Surgery, Faculty of Dentistry, Ankara University, Ankara, Turkey

## Abstract

Objectives: To compare the three non-steroidal anti-inflammatory agents (NSAIDs) diclofenac potassium, etodolac and naproxen sodium in relation to pain, swelling and trismus following impacted third molar surgery. Study Design: The study was a randomized and a double-blinded study which included 42 healthy young individuals with impacted third molars and bone retention. Patients were randomly assigned to 3 groups (n: 14) to which diclofenac potassium, naproxen sodium and etodolac were administered orally an hour before the operation. Impacted third molars were surgically extracted with local anaesthesia. Visual analog scales (VAS) were used to assess the pain in the 6th, 12th hours and on the 1st, 2nd, 3rd, 5th, and 7th days postoperatively. Swelling was evaluated using ultrasound (US) and mouth opening (trismus) was measured with a composing stick pre and post operatively on the 2nd and 7th days respectively. Results: Regarding pain alleviation, diclofenac potassium was better than naproxen sodium and naproxen sodium was better than etodolac but these differences were not statistically significant. US measurements showed that the swelling on postoperative 2nd day was significantly lowest with diclofenac potassium as compared to others (p= 0.027) while naproxen sodium and etodolac acted similarly (p=0.747). No difference was noted regarding trismus in any of the groups. Conclusions: NSAIDs (diclofenac, naproxen and etodolac) are somehow similarly effective for controlling pain and trismus following extraction of mandibular third molars but diclofenac potassium surpasses others in reduction of swelling.

** Key words:**Diclofenac potassium, naproxen sodium, etodolac, impacted third molar surgery, pain, swelling, trismus.

## Introduction

The surgical removal of impacted third molars is one of the most frequently performed procedures in oral surgery and afterwards complications such as post-operative pain, swelling and trismus may occur (1). As prostaglandins are presumed to be primary mediator of acute postsurgical inflammatory changes, these patients, therefore are ideal clinical subjects to study the effect of anti-inflammatory agents on sequelae of teeth extractions such as pain, edema and trismus (2).

Non-steroidal anti-inflammatory drugs (NSAIDs) are regarded as effective medications in the management of pain and other discomforts associated with oral surgery and exert their therapeutic effect through inhibition of cyclooxygenase (COX), which inhibits prostaglandin production whose synergistic interactions with other mediators promote local inflammatory reactions and hyperalgesia. Traditionally, two isoforms of COX are known: COX-1, a constitutive form expressed in almost all tissues which is responsible for the routine physiological functions of prostanoids, including gastric mucosal protection and vascular homeostasis and COX-2, which is found in a limited number of tissues such as kidney, prostate and brain which is primarily responsible for the synthesis of prostanoids and mediation of responses to pathological processes, such as inflammation, pain and fever (2-4). Naproxen sodium, diclofenac potassium and etodolac have both COX-1 and COX-2 inhibitory effects (4).

Naproxen sodium is a propionic acid derivative mainly used in osteoarthritis and rheumatoid arthritis and is also used as an antipyretic and anti-analgesic agent. Naproxen sodium is a NSAID that is traditionally orally administered and the usual doses for oral surgical procedures range between 275 and 550mg (5).

Diclofenac potassium is a NSAID that is either available as an immediate release oral potassium salt tablet form, or as a delayed-release sodium salt tablet form. Many studies showed efficacy of diclofenac as compared to other NSAIDs in management of acute pain following third molar surgery and other dental surgical procedures. As an analgesic and anti-inflammatory agent, diclofenac dose ranges between 25 and 100 mg po for oral surgical procedures (6-9).

Etodolac is a NSAID that is in acetic acid preparation form which is used in treating various acute and chronic musculoskeletal conditions, including osteoarthritis of all joints (10). Earlier studies have reported that etodolac in 50 to 400 mg/day po doses provides dose-related relief of moderate to severe postoperative pain from a variety of surgical and dental procedures (11).

Non-steroidal anti-inflammatory drugs (NSAIDs), when administered pre-operatively, can be absorbed and distributed to oral tissues before the initiation of surgical trauma, thus ensuring a blockade of arachidonic acid pathway, with subsequent reduction in the occurrence of post-operative swelling, trismus, discomfort and pain (12).

The objective of this study is to compare the preemptive administration of diclofenac potassium and etodolac and naproxen sodium on postoperative management of pain, swelling and trismus following removal of impacted mandibular third molars.

## Material and Methods

A clinical, randomized and double-blinded study which included 42 patients was carried out. Healthy outpatients of either gender, between the ages of 17 and 25 years, who presented to the Oral and Maxillofacial Surgery Clinics for surgical removal of mandibular third molars were included in the study. This study lasted a year and a half. The trial was conducted in accordance with the standards of Good Clinical Practices for NSAIDs drugs and guidelines of the Helsinki Declaration on human subjects. Also the local Institutional Review Board approved the design of the study. Exclusion criteria were hepatic or renal disease, blood dyscrasias, gastric ulcer, heart disease, known hypersensitivities, allergies or reactions to any of the study medications, pregnancy and lactation. In addition, patients who had taken any drug within last month before surgery were excluded from the study.

The study protocol was explained to all patients in detail and written informed consent was obtained. Patients were randomly allocated into three groups in a double -blinded fashion by using a prepared drug box with three compartments coded A (etodolac group), B (naproxen sodium group) and C (diclofenac potassium group) and each drug was handed as a coated tablet to patients 1 hour before the operations by the auxiliary personnel. In summary; a total of 42 patients were randomly allocated into 3 groups of 14 third molar teeth as mentioned above (A, B and C groups).

Patients in group A were given etodolac (Tadolak 200 mg tablet, Saba Drug Industries, Turkey). Group B received naproxen so-dium (Apranax 275 mg tablet, Abdi İbrahim Drug Industries, Turkey) and group C was administered diclofenac potassium (Cata-flam 50 mg tablet, Novartis Pharma AG, Switzerland).

The degree of surgical difficulty of impacted third molars was assessed using Archers Classification (Class I and II). Vertical or mesio angulation and bony retention were taken into consideration. Oral perioperative antibiotics (500 mg amoxicilin, Alfoxil 500 mg tablet Abfar Drug Industries, Turkey), were administered to all patients 1 hour before the surgery also chlorhexidine gluconate 0.2% (Klorhex Mouthwash, 200ML, Drogsan Drug Industries, Turkey) was given to all patients as mouth wash. On the following day after surgery, rescue analgesic (Paracatemol 500 mg, Parol tablet, Atabay Drug Industries, Turkey) was made available to all patients for use if needed.

- Surgical procedure

Surgery of the impacted third molars was carried out under local anaesthesia (2% Articain hydrochloride with 1:100.000 adrenaline) with buccal guttering technique after adequate elevation and reflection of buccal mucoperiosteal flap. Tooth delivery was followed by meticulous irrigation of the surgical site with physiologic saline (0,9 %). The three-sided muco-periosteal flap was repositioned and sutured. A single experienced surgeon performed the all surgical procedures. If operation times exceeded 30 minutes, those patients were excluded from the study.

- Measurement of Pain Intensity 

Perioperative pain was assessed using Visual Analogue Scale (VAS). Accordingly, pain was recorded as: ‘no pain’ (no discomfort, VAS: 0), ‘mild pain’ (almost unnoticeable pain, VAS: 1-2), ‘moderate pain’ (noticeable pain but patient can engage in routine daily activities, VAS:3-4), ‘heavy pain’ (very noticeable pain but patient can engage in routine daily activities, VAS: 5-6), ‘severe pain’ (significant pain, patient can perform daily activities, VAS:7-8) and ‘intolerable pain’ (patient cannot perform their daily activities and has to take rescue analgesic medication, VAS:9-10). For each patient, the subjective appropriate score was recorded by a questionnaire at 6th, 12th hours and on 1st, 2nd, 3rd, 4th, 5th and 7th days postoperatively. Before leaving the clinic, the surgeon ensured that all patients were thoroughly instructed on how to complete the pain self-assessment diary and when to take medications.

- Measurement of swelling

The facial swelling was measured quantitatively in milimeters (mm) using ultrasound (US). Ultrasonographic measurements were carried out preoperatively and postoperatively at second and seventh days by single experienced radiologist. The ultrasound examinations were performed using linear 12 MHz probes (Toshiba SSA-770A/80, Aplio, Japan). The patients were placed in supine position and their faces were slightly turned to the contralateral side. The probe was placed transversely on the buccal space and short axis images were obtained. The anterior border of the ramus of the mandible and anterior border of the masseter muscle was used as reference landmarks for standardization of measurements. The probe was oriented transversely so that the cortex of ramus of mandibula lied as a definitive echogenic horizontal line posteriorly on the image screen. Then the vertical distance from the skin to this echogenic line was measured. Measurements were done on the screen after freezing the optimal image and obtaining three consecutive measurements. The median value was recorded and registered to the closest 0.1 mm. Both sides were measured in the same manner. This distance encompassed the skin and the buccal fat pad. This anatomic area was markedly affected by the edema and hemorrhage caused by tooth extraction. Patients were instructed to close their mouths and contact the upper and lower teeth in a natural relaxed way that maintained light interocclusal contact and told not to forcefully clench the jaw, teeth or masticatory muscles during measurements. Care was taken to avoid compression of buccal space.

- Measurement of maximum mouth opening ability (Trismus)

Mouth opening ability was measured in millimetres (mm) with a regular composing stick preoperatively and postoperatively at second and seventh postoperative days. The incisal edge of the maxillary central teeth and incisal edge of mandibular central teeth were used as reference points at the most available maximum mouth opening.

- Statistical Analysis

Data analysis was carried out using commercially available software (Statistical Package for Social Sciences, SPSS for Windows 15.0, Inc, Chicago, Illinois, USA). Comparison of the independent drug groups were carried out using ‘Kruskal Wallis Test’. Double comparisons of the groups were done with ‘Mann-Whitney U Test’. Multiple comparisons of the data from different re-lated times among drug groups were calculated using ‘Friedman Test’ and double comparisons of the same data were studied with ‘Wilcoxon Signed Ranks Test’. The level of significance was set at P<0.05.

Results

**Figure 1:**
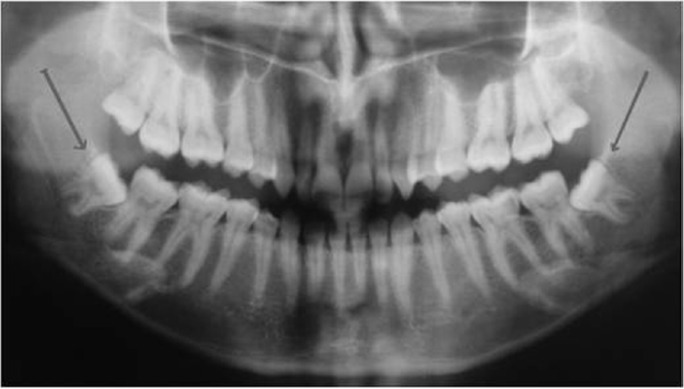
A panoramic roentgen of a patient (MD) operated for removal of mandibular third molars. The classifications of third molars were compatible with Class II, in Mesio-Angular position according to Archer Third Molar Classification.

A total of 42 patients (14 male and 28 female; age ranging from 18 to 25 year; mean age, 20.8± 4.1 year), who had impacted molars which were Class I or Class II and in vertical or mesio-angular positions as described in Archer’s Impacted Third Molar Classification (Fig. 1) were included in this study. The study was carried out in a prospective, randomized and double-blinded manner.

The average surgery time was 21.5 minutes. No adverse effects or complications related to surgery were recorded in any treatment group throughout the study period. No alterations of the mandibular nerve conductivity were recorded. The lip and cheek sensitivity returned to normal in all patients within 2 to 4 hours postoperatively.

- Postoperative pain assessment

Collected pain data was assessed with descriptive, multiple and double comparative statistical analysis. All drug groups (Etodolac-A, Naproxen Sodium-B and Diclofenac Potassium-C) were compared with each other for pain intensity at predetermined times by using Kruskal Wallis Test ( Table 1) and results were not statistically significant (P>.05).

**Table 1:**
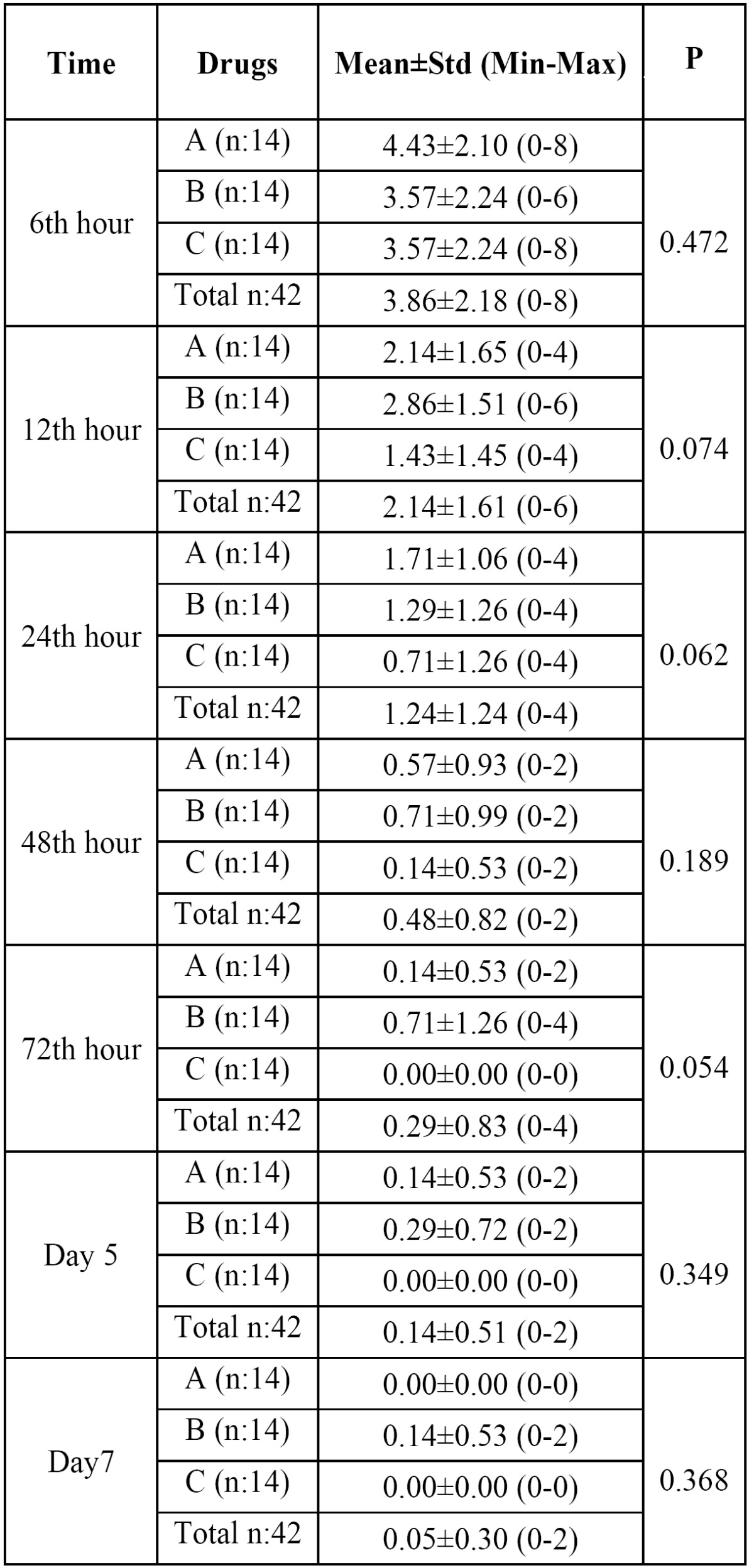
Postoperative pain intensity values measured by VAS (Visual analogue scale) post surgery (postoperative 6th hour to day 7). A: Etodolac, B: Naproxen Sodium, C: Diclofenac Potassium, n: Number of subjects, Std: Standart deviation, Min: Minimum, Max: Maximum.

**Figure 2:**
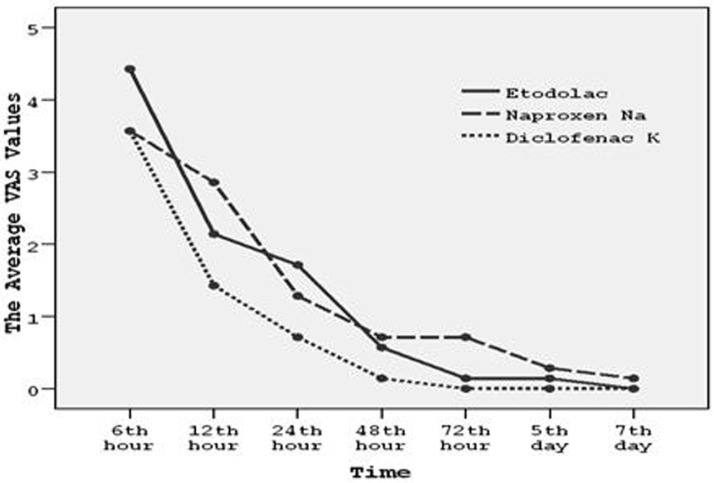
The efficacies of drugs on pain depicted in a time course.

The efficacy of drugs on pain according to time course was also shown graphically in figure 2. Diclofenac potassium appeared to be the most effective of all in this regard but results were not statistically significant (P>.05).

- Postoperative swelling assessment

Differences among groups were compared using ‘Kruskal Wallis Test in terms of efficacy on swelling ( Table 2). Results showed that;

**Table 2:**
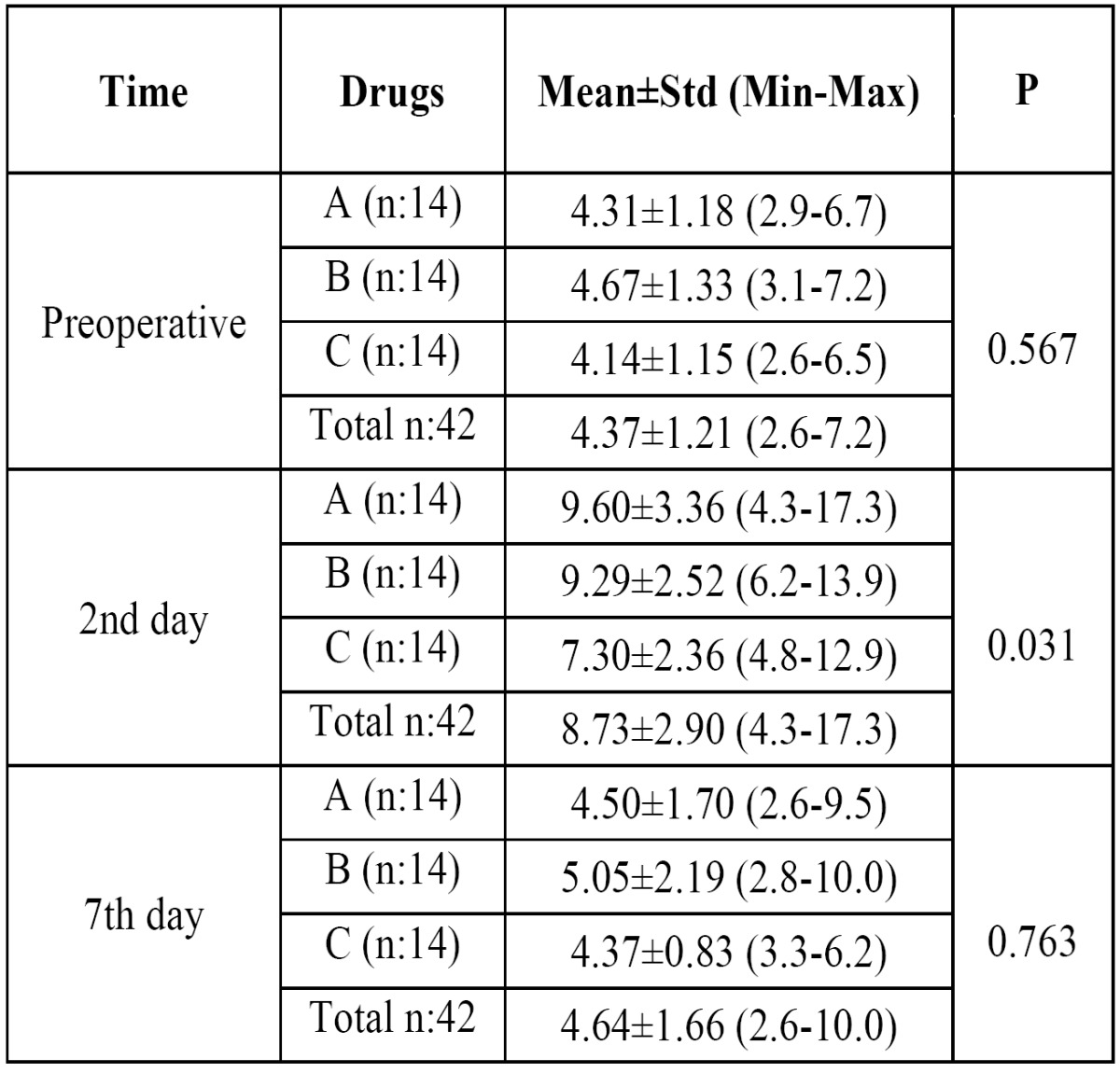
Postoperative edema measured by ultrasound (buccal area thickness in milimeters) preoperatively, on the 2nd day and on the 7th day following molar surgery A: Etodolac, B: Naproxen Sodium, C: Diclofenac Potassium, n: Number of subjects, Std: Standart deviation, Min: Minimum, Max: Maximum.

1- There was no significant difference between the drug groups at preoperative and postoperative 7th days (P>.05).

2- Significant differences were detected at postoperative 2th day in terms of efficacy on swelling (P<.05).

Double comparison of drug groups was performed by using ‘Mann-Whiney U Test’ at 2th day. Results showed that:

1- When Etodolac and Naproxen sodium were compared, in terms of efficacy on swelling, there was no significant difference (P=, 747).

2- Diclofenac potassium was significantly more effective on swelling than Etodolac and Naproxen sodium respectively (P=.027) (P=.020).

**Figure 3:**
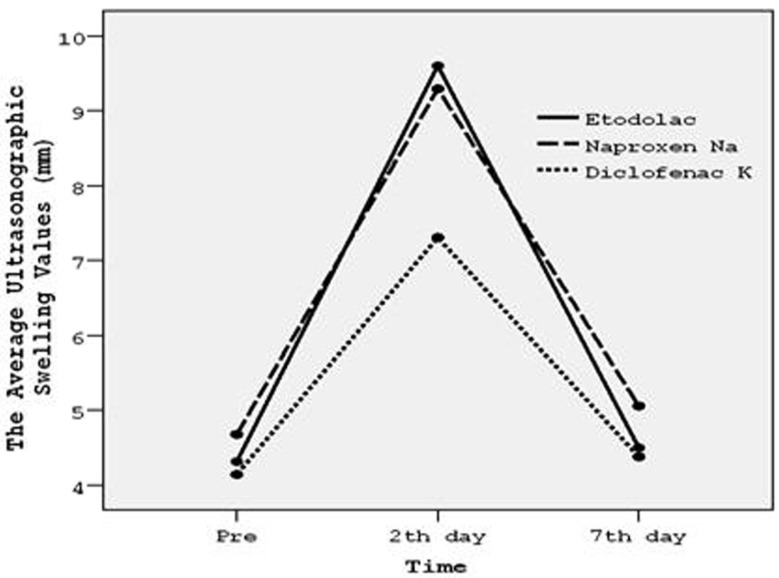
Graphic shows the efficacies of drugs on swelling versus time.

The efficacy of drug groups on swelling according to time courses was shown graphically in figure 3. Diclofenac potassium was more effective than etodolac and naproxen sodium and this effect was most significant at postoperative 2th day (P<.05).

- Postoperative mouth opening ability (Trismus) assessment

Mouth opening ability was used to denote trismus and decreased postoperatively. Results (Kruskal Wallis test) showed that there were no statistical differences between the drug groups regarding trismus (P>.05) ( Table 3).

**Table 3:**
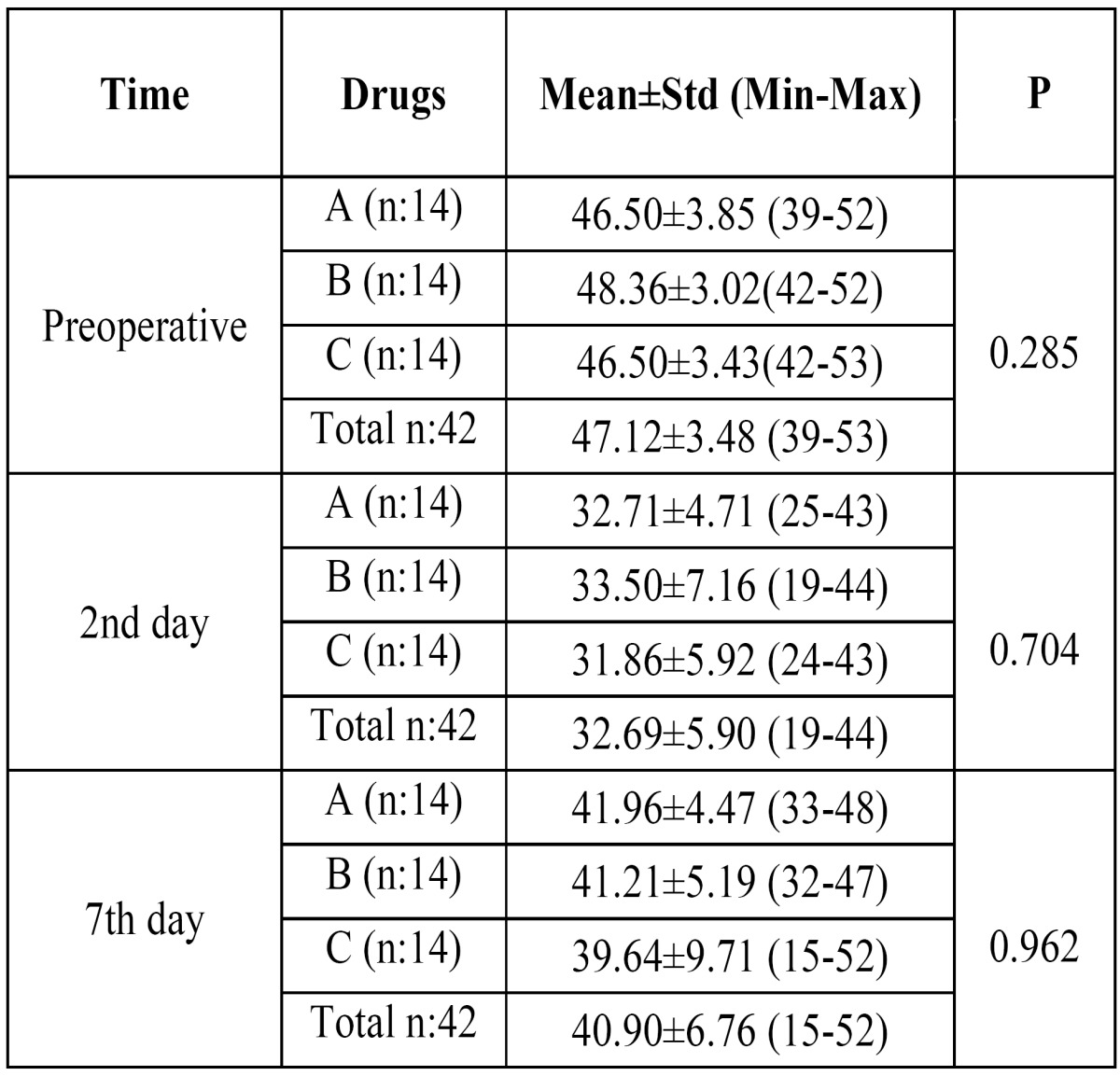
Postoperative trismus measurements (mouth opening) in milimeters preoperatively, on the 2nd day and on the 7th day following molar surgery. A: Etodolac, B: Naproxen Sodium, C: Diclofenac Potassium, n: Number of subjects, Std: Standart deviation, Min: Minimum, Max: Maximum.

**Figure 4:**
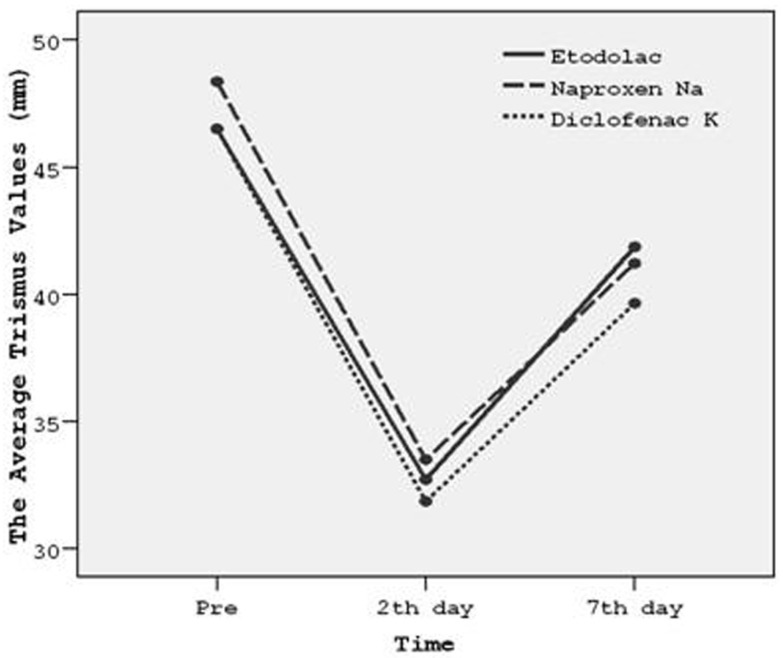
The efficacies of drugs on average trismus values presented over a time course.

The efficacy of drugs on mouth opening ability according to the postoperative time course was also graphically shown (Fig. 4). Results showed that diclofenac potassium was more effective than etodolac and naproxen sodium. However findings were not statistically significant as mentioned above (P>.05).

None of the patients in any of the groups used the rescue analgesic although it was made available to them. No major or minor complications related to medications were encountered in any of the groups.

## Discussion

In the present study, the efficacy of NSAIDs which were diclofenac potassium, naproxen sodium and etodolac were investigated in regards to pain, swelling and trismus following third molar surgery. To our knowledge, there is no comparative study comparing these three drugs on this subject.

Close relation between postoperative complications (pain,swelling and trismus) and operation time was shown in various studies (13). In the present study, any third molar operation that exceeded 30 minutes was excluded from the study. If there was any deviation from the surgical technique described in the material and methods section due to difficulties in extraction, these patients were excluded as well.

Timing of NSAID administration is a subject of debate. Some authors favour preoperative medication arguing that it allows better postoperative analgesia control by suppression of peripheral and central sensitization while others argue that preemptive medication performs less compared to postoperative administration of the same drugs (12). In the study by Bridgman et al. diclofenac sodium did not demonstrate any significant pre-emptive analgesic effects (14). On the contrary Shah et al. compared the analgesic effect of diclofenac sodium before and after third impacted molar surgery and suggested that preemptive medication provides better pain control (15). The findings in our study also support the notion to administer drugs preoperatively.

In this study, diclofenac potassium was slightly more effective on postoperative pain but the difference between diclofenac, etodolac and naproxen regarding pain in any postoperative time period was not statistically significant (Fig. 2). Oncul et al. compared preoperative IV diclofenac sodium with IV paracetamol (acetaminophen) and Lornoxicam and concluded that although all were equally effective on postoperative pain (16). Diclofenac was reported to be better in controlling postoperative pain compared to tramadol and ketorolac but less compared to piroxicam and nimesulid and tenoxicam (17-21).

There are only a few studies about the efficacy of Etodolac on molar teeth extraction surgery. Etodolac performed better compared to placebo and showed similar efficacy compared to aspirin (22), acetaminophen plus codeine combination and zomepirac regarding postoperative pain but performed less compared to diflunisal (23,24).

Combination of NSAID’s with steroids appears to be more effective in controlling complications after third molar surgery as compared to NSAID’s alone especially regarding pain and swelling, but carry a certain risk of adversity such as adrenal suppression and concerns about impaired wound healing, therefore their routine use has not been justified (8). Bamgbose et al. compared co-administration of dexamethasone and diclofenac potassium with diclofenac potassium alone. They concluded that co-administration was more effective as compared to diclofenac potassium alone for pain and swelling but no significant difference was noted regarding trismus (8). Likewise, Lopez-Carriches et al. compared diclofenac to metilprednisolone and steroids slightly performed better in pain reduction and swelling but not in trismus (9).

Bjørnsson et al. compared naproxen and acetaminophen on postoperative sequelae after impacted third molar surgery. They found that naproxen was more effective on pain than acetaminophen, but acetaminophen was more effective on swelling than naproxen (5). In the present study diclofenac appeared more effective on swelling than naproxen. When acetaminophen and diclofenac are compared, they were reported to have similar efficacy (16,25). Acetaminophen is considered safer and combination with diclofenac is reported to perform better compared to diclofenac alone and acetaminophen codeine combination (25,26).

**Figure 5:**
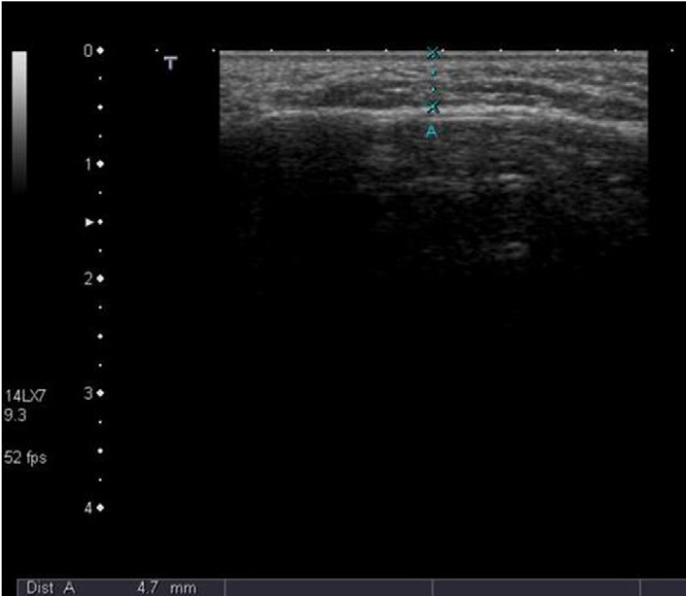
Evolution of postoperative swelling. Preoperatively right buccal thickness measures 4,7 mm.

**Figure 6:**
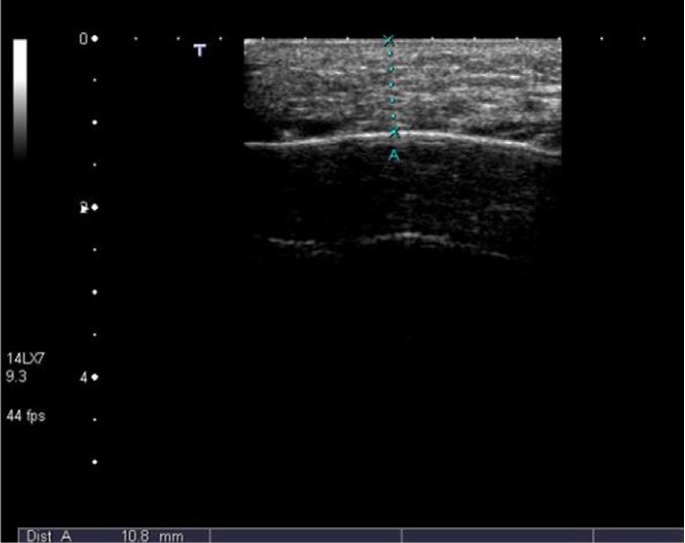
Measurement on 2nd postoperative day reveals marked soft tissue swelling of 10,8 mm.

**Figure 7:**
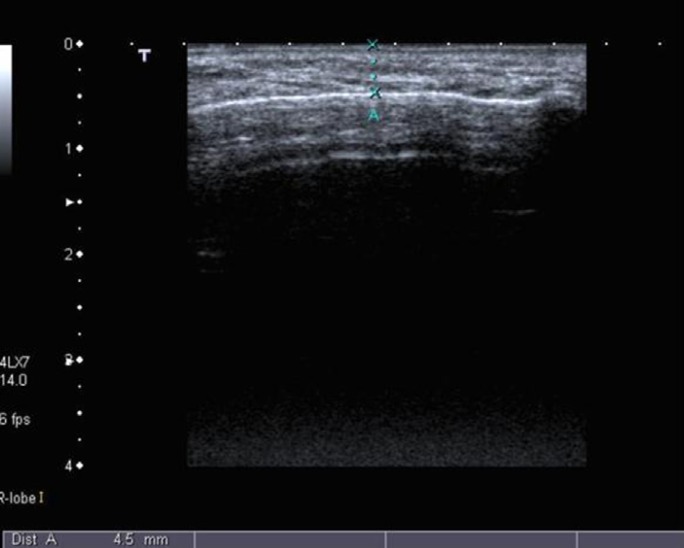
Measurement on 7th postoperative day showed that the soft tissue swelling has regressed markedly back to 4.5 mm.

Several methods were employed to measure postoperative swelling including VAS, stereophotography, sutures and face-bow (13,27-29). Ultrasound method was utilized in a few studies and reported to be an efficacious method of measurement and as good as CT (30). Ultrasound was used this study to measure postoperative swelling (Fig. 5-7).

In the present study, there were no differences between the drug groups on postoperative mouth opening ability (trismus). If the time courses are surveyed in the present study, a little advanced effect of diclofenac may be encountered as compared to other drugs but the difference was not statistically significant. Many studies support the present study in terms of mouth opening ability (5,8).

In conclusion, in the present study; diclofenac potassium performed significantly better on swelling and as good as other two drugs naproxen sodium and etodolac regarding pain and trismus. In other words, the present study aligned the drugs as diclofanac potassium> naproxen sodium = etodolac in terms of reducing the postoperative third molar surgery sequelae. Various methods, such as different NSAIDs, laser, steroids etc. were tested and will continue to be tested on this issue.

## References

[1] Blondeau F, Daniel NG (2007). Extraction of impacted mandibular third molars: postoperative complications and their risk factors. J Can Dent Assoc.

[2] Morrison BW, Fricke J, Brown J, Yuan W, Kotey P, Mehlisch D (2000). The optimal analgesic dose of rofecoxib: overview of six randomized controlled trials. J Am Dent Assoc.

[3] Barden J, Edwards JE, McQuay HJ, Wiffen PJ, Moore RA (2004). Relative efficacy of oral analgesics after third molar extraction. Br Dent J.

[4] Lee Y, Rodriguez C, Dionne RA (2005). The role of COX-2 in acute pain and the use of selective COX-2 inhibitors for acute pain relief. Curr Pharm Des.

[5] Bjørnsson GA, Haanaes HR, Skoglund LA (2003). Naproxen 500 mg bid versus acetaminophen 1000 mg qid: effect on swelling and other acute postoperative events after bilateral third molar surgery. J Clin Pharmacol.

[6] Zuniga JR, Phillips CL, Shugars D, Lyon JA, Peroutka SJ, Swarbrick J (2004). Analgesic safety and efficacy of diclofenac sodium softgels on postoperative third molar extraction pain. J Oral Maxillofac Surg.

[7] Esteller-Martínez V, Paredes-García J, Valmaseda-Castellón E, Berini-Aytés L, Gay-Escoda C (2004). Analgesic efficacy of diclofenac sodium versus ibuprofen following surgical extraction of impacted lower third molars. Med Oral Patol Oral Cir Bucal.

[8] Bamgbose BO, Akinwande JA, Adeyemo WL, Ladeinde AL, Arotiba GT, Ogunlewe MO (2005). Effects of co-administered dexamethasone and diclofenac potassium on pain, swelling and trismus following third molar surgery. Head Face Med.

[9] López-Carriches C, Martínez-González JM, Donado-Rodríguez M (2005). Analgesic efficacy of diclofenac versus methylprednisolone in the control of postoperative pain after surgical removal of lower third molars. Med Oral Patol Oral Cir Bucal.

[10] Veys EM (1994). Clinical performance of etodolac in patients with osteoarthiritis and rheumatoid arthiritis. Eur J Rheumatol Inflamm.

[11] Koseoglu BG, Ozturk S, Kocak H, Palanduz S, Cefle K (2008). The effects of etodolac, nimesulid and naproxen sodium on the frequency of sister chromatid exchange after enclused third molars surgery. Yonsei Med J.

[12] Savage MG, Henry MA (2004). Preoperative nonsteroidal anti-inflammatory agents: review of the literature. Oral Surg Oral Med Oral Pathol Oral Radiol Endod.

[13] Absi EG, Shepherd JP (1993). A comparison of morbidity following the removal of lower third molars by the lingual split and surgical bur methods. Int J Oral Maxillofac Surg.

[14] Bridgman JB, Gillgrass TG, Zacharias M (1996). The absence of any pre-emptive analgesic effect for non-steroidal anti-inflammatory drugs. Br J Oral Maxillofac Surg.

[15] Shah R, Mahajan A, Shah N, Dadhania AP (2012). Preemptive analgesia in third molar impaction surgery. Natl J Maxillofac Surg.

[16] Tuzuner Oncul AM, Yazicioglu D, Alanoglu Z, Demiralp S, Ozturk A, Ucok C (2011). Postoperative analgesia in impacted third molar surgery: the role of preoperative diclofenac sodium, paracetamol and lornoxicam. Med Princ Pract.

[17] Pandit MK, Godhi S, Lall AB (2011). Preoperative intravenous tramadol versus diclofenac for preventing postoperative pain after third molar surgery: a comparative study. J Maxillofac Oral Surg.

[18] Christensen K, Daniels S, Bandy D, Ernst CC, Hamilton DA, Mermelstein FH (2011). A double-blind placebo-controlled comparison of a novel formulation of intravenous diclofenac and ketorolac for postoperative third molar extraction pain. Anesth Prog.

[19] Mohammad S, Singh V, Wadhwani P, Tayade HP, Rathod OK (2012). Sublingual piroxicam in the management of postoperative pain after surgical removal of impacted mandibular third molar. Indian J Dent Res.

[20] Levrini L, Carraro M, Rizzo S, Salgarello S, Bertelli E, Pelliccioni GA (2008). Prescriptions of NSAIDs to patients undergoing third molar surgery: an observational prospective, multicentre survey. Clin Drug Investig.

[21] Roelofse JA, Van der Bijl P, Joubert JJ (1993). An open comparative study of the analgesic effects of tenoxicam and diclofenac sodium after third molar surgery. Anesth Pain Control Dent.

[22] Hutton CE (1983). The effectiveness of 100 and 200 mg etodolac (Ultradol), aspirin, and placebo in patients with pain following oral surgery. Oral Surg Oral Med Oral Pathol.

[23] Comfort MB, Tse AS, Tsang AC, McGrath C (2002). A study of the comparative efficacy of three common analgesics in the control of pain after third molar surgery under local anaesthesia. Aust Dent J.

[24] Scott R, Ellis E, Upton LG (1986). Double-blind evaluation of etodolac (200 mg, 400 mg) compared with zomepirac (100 mg) and placebo on third molar extraction pain. Oral Surg Oral Med Oral Pathol.

[25] Breivik EK, Barkvoll P, Skovlund E (1999). Combining diclofenac with acetaminophen or acetaminophen-codeine after oral surgery: a randomized, double-blind single-dose study. Clin Pharmacol Ther.

[26] Ervens K, Schiffmann L, Berger G, Hoffmeister B (2004). Colon perforation with acute peritonitis after taking clindamycin and diclofenac following wisdom tooth removal. J Craniomaxillofac Surg.

[27] Burke PH, Banks P, Beard LF, Tee JE, Hughes C (1983). Stereophotographic measurement of change in facial soft tissue morphology following surgery. Br J Oral Surg.

[28] Gallardo F, Carstens M, Ayarza M (1990). Analgesic and antiinflammatory effects of glucamethacin (a nonsteroidal antiinflammatory analgesic) after the removal of impacted third molars. Oral Surg Oral Med Oral Pathol.

[29] Krekmanov L, Nordenram A (1986). Postoperative complications after surgical removal of mandibular third molars. Effects of penicillin V and chlorhexidine. Int J Oral Maxillofac Surg.

[30] Esen E, Taşar F, Akhan O (1999). Determination of the anti-inflammatory effects of methylprednisolone on the sequelae of third molar surgery. J Oral Maxillofac Surg.

